# Environmental Temperature Affects Prevalence of Blood Parasites of Birds on an Elevation Gradient: Implications for Disease in a Warming Climate

**DOI:** 10.1371/journal.pone.0039208

**Published:** 2012-06-19

**Authors:** Itzel Zamora-Vilchis, Stephen E. Williams, Christopher N. Johnson

**Affiliations:** Centre for Tropical Biodiversity and Climate Change, School of Marine and Tropical Biology, James Cook University, Townsville, Queensland, Australia; University of Georgia, United States of America

## Abstract

**Background:**

The rising global temperature is predicted to expand the distribution of vector-borne diseases both in latitude and altitude. Many host communities could be affected by increased prevalence of disease, heightening the risk of extinction for many already threatened species. To understand how host communities could be affected by changing parasite distributions, we need information on the distribution of parasites in relation to variables like temperature and rainfall that are predicted to be affected by climate change.

**Methodology/Principal Findings:**

We determined relations between prevalence of blood parasites, temperature, and seasonal rainfall in a bird community of the Australian Wet Tropics along an elevation gradient. We used PCR screening to investigate the prevalence and lineage diversity of four genera of blood parasites (*Plasmodium*, *Haemoproteus*, *Leucocytozoon* and *Trypanosoma*) in 403 birds. The overall prevalence of the four genera of blood parasites was 32.3%, with *Haemoproteus* the predominant genus. A total of 48 unique lineages were detected. Independent of elevation, parasite prevalence was positively and strongly associated with annual temperature. Parasite prevalence was elevated during the dry season.

**Conclusions/Significance:**

Low temperatures of the higher elevations can help to reduce both the development of avian haematozoa and the abundance of parasite vectors, and hence parasite prevalence. In contrast, high temperatures of the lowland areas provide an excellent environment for the development and transmission of haematozoa. We showed that rising temperatures are likely to lead to increased prevalence of parasites in birds, and may force shifts of bird distribution to higher elevations. We found that upland tropical areas are currently a low-disease habitat and their conservation should be given high priority in management plans under climate change.

## Introduction

Many studies have described trends in the structure of assemblages along elevational gradients, and have found temperature to be one of the main variables controlling elevational distribution across a diverse taxonomic and ecological range of species [1]–[3]. However, little is known about the distribution of pathogenic organisms on these gradients. Vector-borne diseases are widely distributed pathogens transmitted to hosts by arthropod vectors such as biting flies [4]. The rising global temperature is predicted to expand the distribution of vector-borne diseases [5]. There are two reasons for this: abundances of most vectors are positively related to temperature [6]; and for most vector-borne diseases, transmission may be enhanced by higher ambient temperature. The development of *Plasmodium*, for example, can occur between 16–30°C, with optimal temperatures around 28–30°C, whereas temperatures lower than 16°C greatly inhibit parasite development [7].

In contrast to predictions for vector borne parasites, many studies have reported reductions in geographical range size and abundance, and shifts to lower latitudes or high altitudes, in a wide range of organisms that are potential hosts for these parasites [8]–[11]. Range expansion of vector borne parasites may increase their prevalence in many host populations. Increased parasite loads can have negative effects on host populations, reducing growth and causing higher mortality and/or lower birth rates [12]–[15]. These effects could amplify the risk of extinction for many already threatened species. The study of parasite distributions in relation to climate gradients is important in helping us to understand how host species might be affected by changing parasite prevalence under climate change. Elevational gradients provide an excellent framework for such research, because temperature is closely related to elevation and elevation differences can cause large changes in temperature over short geographic distances [16].

The main aim of this study was to determine how temperature and rainfall influence prevalence of blood parasites in tropical birds. To do this we studied bird communities along elevation gradients in the Australian Wet Tropics. This bioregion is one of the best-studied tropical rainforests in the world. It consists of a strip of coastal plains and a series of adjacent mountain systems, with an altitude range from sea level to 1600 meters above sea level [17]. Species distribution models predict that under impending temperature rises many bird species in this region could experience significant range reductions, increased population fragmentation and declines in population size, and therefore heightened risk of extinction [18]–[21]. However, there has been no study of elevational distribution of bird parasites and how climate change could affect their prevalence.

We present data on the prevalence and lineage diversity of four genera of blood parasites (*Plasmodium*, *Haemoproteus*, *Leucocytozoon* and *Trypanosoma*) in birds of the Australian Wet Tropics in relation to elevation. We test for relations between parasite prevalence, elevation, temperature, and seasonal rainfall. These studies are not only important to implement future models on how increase of temperature will affect parasite loads but also how host communities could be affected by parasites.

## Methods

### Ethics Statement

This study was carried out under permits WISP01559303 and WITK01559403 of the Environmental Protection Agency, Queensland Parks and Wildlife Service, Australia. This research was approved by the Committee on the Animal Ethics of the James Cook University (Permit Number A-1120). Birds were caught and banded under the license number 2664 from the Department of the Environment and Heritage, Australia. All birds were released after blood samples were taken.

### Study Area and Bird Community

The Australian Wet Tropics bioregion (AWT) is located in far North Queensland between 19°30’S and 15°30’S. The region is dominated by tropical rainforest, which covers an area of 10,000 km^2^ and is primarily distributed along the mountain ranges [17]. In this region, temperature is one of the most important variables driving trends of distribution of many species along elevational gradients of the mountain systems [22]. Mean annual rainfall in the region varies between 1500 mm and 3300 mm [21], with approximately 75–90% falling between November and April [17]. The bird community shows strong trends of assemblage structure along the elevational gradient with high levels of regional endemism in the uplands [18], [23]. Both species richness and bird abundance exhibit a humped-shaped pattern with elevation, with highest values found between 600 m and 800 m [22].

### Data Collection

**Figure 1:**
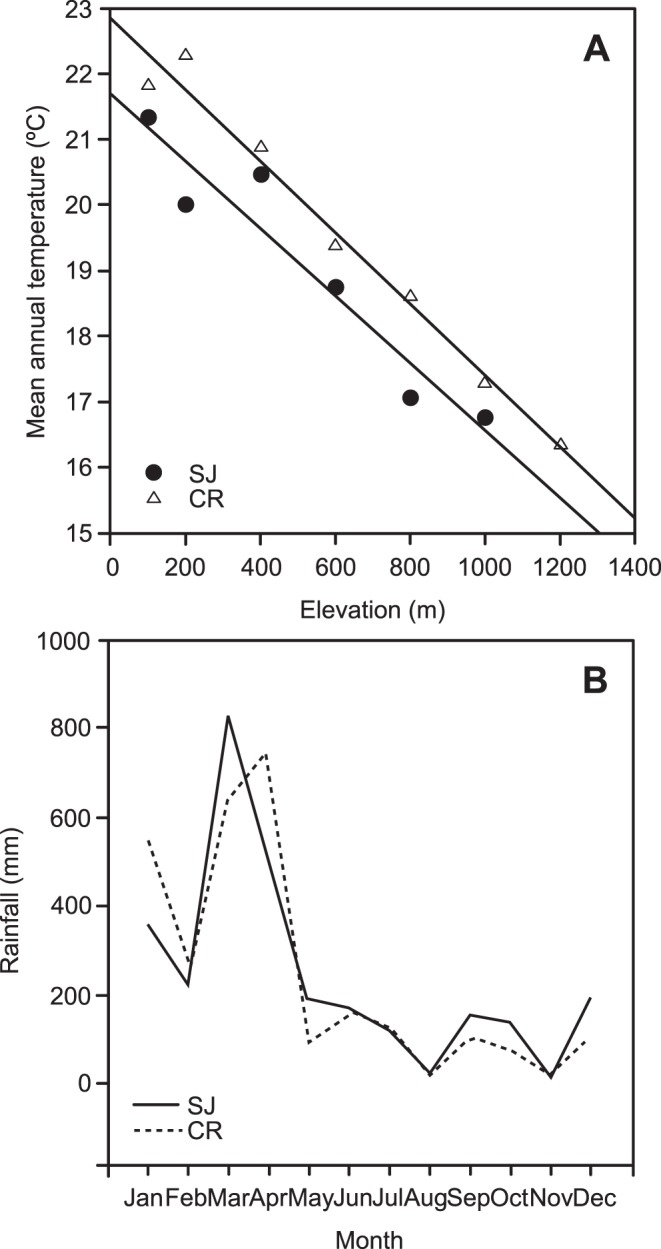
Variation of temperature and rainfall at the AWT. A) Predicted variation of Mean annual temperature as a function of elevation. Temperature decreased at an approximately rate of 1°C per 200 m altitude and there was approximately 1°C difference between the two localities sampled at the same elevation and B) Monthly variation of rainfall at the two localities within the region indicated that the dry season began on May and was extended and acute through November or December when the rainy season began. The highest values of rainfall were between February and May. Localities: South Johnston (SJ) and Carbine Range (CR).

Data were collected during 2005 and 2006 from two localities of the region: the South Johnston/Atherton Tablelands area (17.62°S; 145.72°E) and the Carbine Range (Lat; Long 16.56°S; 145.28°E). These localities are around 125 km apart. Nevertheless, they are within the same bioregion and have similar vegetation structure and almost identical bird faunas [22], [24]. There is a strong relationship with bird assemblages across elevation in the two localities, and that relationship is similar in both [22]. Bird blood samples were collected at different elevation sites (Table 1). Mean annual temperature for each elevational site located at every 200 m of elevation in each locality was measured using data loggers maintained by the Centre for Tropical Biodiversity and Climate Change at James Cook University. Each logger consists of five sensors, which measure air temperature, relative humidity, soil moisture, soil temperature, and condensation at 15 min intervals. Mean monthly rainfall for each elevational site at each locality was estimated using daily rainfall data extracted from the Australian Water Availability Project http://www.bom.gov.au/jsp/awap/. Temperature decreased at an approximate rate of 1°C per 200 m altitude and there was approximately 1°C difference between the two areas sampled at the same elevation (Figure 1A). The monthly average rainfall indicated that the dry season began in May and was extended and acute until November or December when the rainy season began. The highest values of rainfall were between February and May (Figure 1B).

**Table 1:** Localities of sampling in the AWT.

Localities	Elevation (m)	MAT (°C)	No. of sampled birds
Carbine Range	100	21.8	14
Carbine Range	400	20.9	27
South Johnston	400	20.5	102
South Johnston	800	17.1	18
Carbine Range	1000	17.3	190
Carbine Range	1200	16.4	52

The elevation, Mean Annual Temperature (MAT) and Number of sample birds for each locality are indicated.

### Study Species

We collected blood samples from 403 individual birds belonging to 40 species in sixteen different families: Acanthizidae, Alcedinidae, Climacteridae, Columbidae, Dicaeidae, Dicruridae, Estrildidae, Eupetidae, Meliphagidae, Muscicapidae, Nectariniidae, Pachycephalidae, Paradisaeidae, Petroicidae, Ptilonorhynchidae and Zosteropidae (all species are listed in Table S1). None of the bird species used here migrate to different geographic regions, and they show specific trends of distribution along the elevation gradient [22], [25]. Birds were caught in mist nets, and approximately 50 to 75 µl of blood was collected by puncture of the brachial vein. Blood samples were stored in Queens lysis buffer [26] for subsequent analysis.

### Molecular Analyses

DNA was extracted from all samples using silica fines [27]. Two nested-PCR protocols were used to detect four genera of blood parasites: one nested PCR assay for *Plasmodium, Haemoproteus* and *Leucocytozoon* targeting a 478 bp section of the mitochondrial cytochrome b gene [28], and another assay for *Trypanosoma* targeting a 326 bp section of 18 S rRNA gene (18 S) [29]. These nested-PCR protocols are highly repeatable and provide significantly higher detection success than inspection of blood smears [28], [29]. For *Plasmodium, Haemoproteus* and *Leucocytozoon* the first PCR step was carried out in a 10 µl reaction, using approximately 50 ng of DNA, 1x GoTaq Green Master Mix (Promega) and 0.5 of each primer (Table S2). Cycling conditions included an initial denaturation step at 94°C for 3 min, followed by 20 cycles of 30 s at 94°C, 30 s annealing at 50°C and 45 s extension at 72°C; and a final extension step of 10 min at 72°C. PCR products from the first reaction were used as a template for two other reactions: one that amplifies specific cytochrome b sequences for the genera *Plasmodium* and *Haemoproteus*, and another for *Leucocytozoon*. Reactions were carried out in a 25 µl volume containing 1x GoTaq Green Master Mix, 0.6 µM of each of the respective primers (Table S2) and 2 µl of the PCR product from the initial reaction. Cycling conditions were identical to the first PCR but performed for 35 cycles instead of 20. The first reaction for *Trypanosoma* was carried out in a 10 µl volume containing 1x GoTaq Green Master Mix, 0.5 µM of each primer (Table S2) and approximately 50 ng of template DNA. Cycling conditions included an initial denaturation at 95°C for 5 min followed by five cycles at 95°C for 1 min, 45°C for 30 s, 65°C for 1 min, and 35 cycles at 95°C for 1 min, 50°C for 30 s, 72°C for 1 min; and a final extension at 65°C for 10 min. The second reaction included 1x GoTaq Green Mastermix, 0.6 µM of each primer (Table S2) and 1 µl of PCR product from the initial reaction. Cycling conditions included an initial denaturation at 96°C for 3 min, followed by 35 cycles at 96°C for 30 s, 63°C for 1 min, 72°C for 30 s and a final extension at 74°C for 7 min. To identify parasite lineages, all the positive products were bidirectionally sequenced. Sequences were edited and aligned using the program Sequencher 4.8. We identified lineages based on single base pair difference. Sequences were deposited in both MalAvi database [30] http://mbio-serv4.mbioekol.lu.se/avianmalaria and GenBank (Accession numbers JX021535-JX021582).

## Results

### Prevalence of Parasites

Of the 403 individual birds screened, 130 (32.3%) tested positive for one or more parasite genera. The predominant parasite was *Haemoproteus* with 80 infected birds (19.9%). *Trypanosoma* and *Leucocytozoon* showed very similar prevalence with 28 (6.9%) and 25 (6.2%) infected birds respectively, whereas *Plasmodium* was present in only 7 (1.7%) birds. An additional 15 (3.7%) individuals were infected with *Haemoproteus* and/or *Plasmodium* but the parasite could not be identified to genus due to low PCR amplification, poor-quality sequence or unresolved multiple infections. Among well-sampled host families (i.e. >15 individuals sampled per family, Table 2), prevalence of *Haemoproteus* ranged from 2.1% (Estrildidae) to 60.3% (Petroicidae). The family with the highest prevalence of *Plasmodium* and *Trypanosoma* was Pachycephalidae with 3.1% and 15.6% respectively, whereas Dicruridae had the highest prevalence of *Leucocytozoon* with 16.3%. Prevalence of the four genera of parasites was similar across different host families.

**Table 2:** Parasite prevalence across host families.

	% of total			Unknown		
Host family	infected birds	*% Hae*	*% Pla*	*% Hae and/or Pla*	*% Leu*	*% Try*
1. Petroicidae	76.7	65.8	1.4	6.8	0	11
2. Pachycephalidae	43.8	31.3	3.1	3.1	0	15.6
3. Dicruridae	30.2	9.3	0	2.3	16.3	11.6
4. Meliphagidae	22.1	4.4	2.9	5.9	10.3	4.4
5. Acanthizidae	15.3	8.1	1.8	0.9	0.9	2.7
6. Estrildidae	8.3	2.1	0	4.2	4.2	0
7. Others	42.9	17.9	3.6	3.6	28.6	14.3
Total	32.3	19.9	1.7	3.7	6.2	6.9

Parasite prevalence of well represented families (1–6; >15 individuals) and other families (7; <15 individuals). Percentage of total number of birds infected and number of birds infected by each parasite genus (%) (*Hae: Haemoproteus, Pla: Plasmodium*, Unknown: either *Haemoproteus* and/or *Plasmodium*, *Leu: Leucocytozoon* and *Try: Trypanosoma*).

### Lineage Diversity

A total of 48 unique lineages of parasites (including the four genera) was detected. *Haemoprotueus* was the genus exhibiting the highest number of lineages (30). *Trypanosoma* and *Leucocytozoon* presented 7 and 6 haplotypes respectively. Finally, for *Plasmodium* only 5 unique lineages were detected (MalAvi lineage names and GenBank accession numbers are listed in Table S3). We found that the four genera were generalist, strictly speaking, as most of the lineages were found in more than one host species. However, most of the *Haemoproteus* lineages were partially specific to host family.

Analysis of parasite lineages along the elevation gradient showed that most of the lineages were present only in certain elevation sites. This was probably due to the observed high lineage diversity and the specific trends of host distribution along the gradient. Only a few lineages of two well-represented families (Petroicidae and Pachycephalidae) were distributed along the entire gradient. Nevertheless, due to the great diversity of lineages found, sample sizes of each of these well-distributed lineages are not large enough to determine significant trends of distribution in relation to elevation, temperature or rainfall.

### Temperature and Prevalence of Bird Blood Parasites on an Elevation Gradient

**Figure 2:**
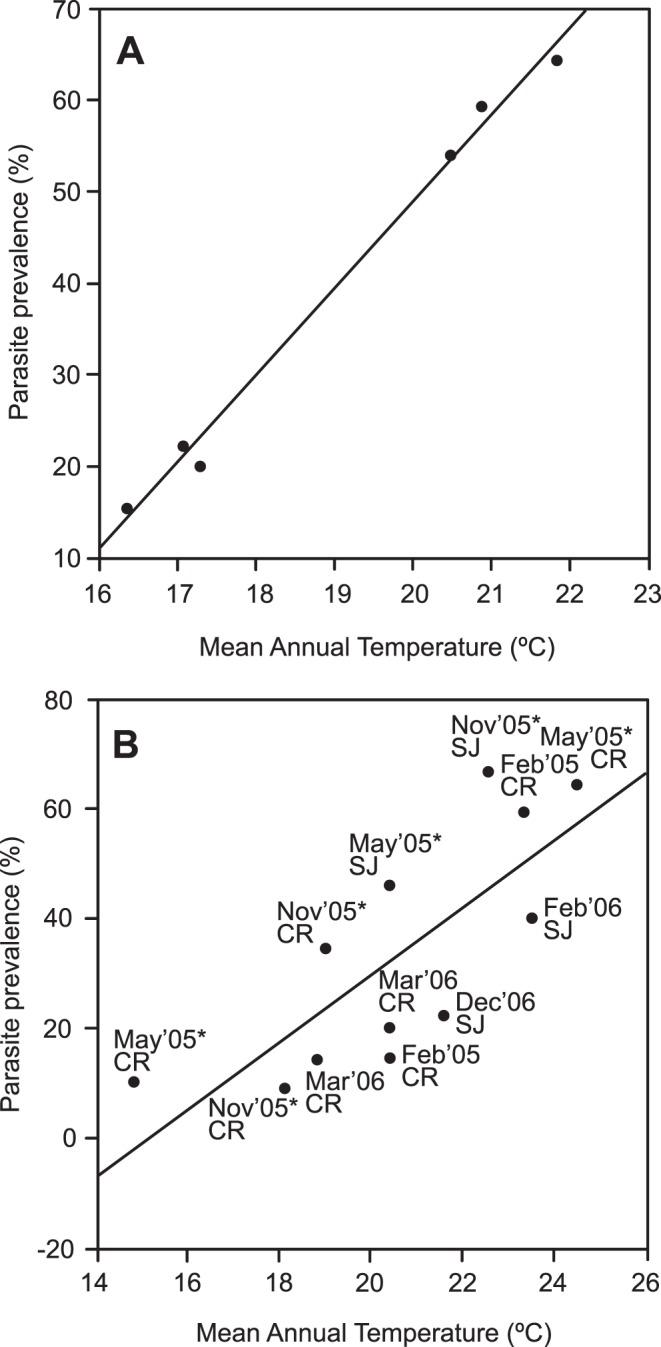
Relationship between overall parasite prevalence and temperature. Predicted variation of overall parasite prevalence as a function of a) Mean annual temperature and b) Mean monthly temperature. Month, year and locality (SJ  =  South Johnston and CR  =  Carbine Range) are indicated for each point. Dry season months are marked with asterisks.

The overall prevalence of infection (of all four parasite genera) was negatively related to elevation (F_1,4_ = 52.45, P<0.002, R^2^ = 0.93) and positively to mean annual temperature (F_1,4_ = 438.98, P<0.00003, R^2^ = 0.99; Figure 2A). A multiple regression model of parasite prevalence on both elevation and temperature was highly significant (F_2,3_ = 164.63, P<0.02, R^2^ = 0.99, Adjusted R^2^ = 0.98) but only temperature contributed significantly to the model (temperature: Beta  = 0.99 P = 0.02; elevation: Beta  = 0.004 P>0.98). We checked for relationships of parasite prevalence to host characteristics, including each species’ geographic range size, body mass and body size, but found no significant relationships (Table S4; only species with more than 5 individuals were used in the analysis).

Relationships of overall parasite prevalence to temperature in well sampled families (represented by >15 individuals and sampled from at least 3 elevations) were positive in Acanthizidae (F_1,3_ = 10.67, P<0.05, R^2^ = 0.78) and Dicruridae (F_1,3_ = 14.53, P<0.05, R^2^ = 0.83), whereas Meliphagidae (F_1,2_ = 2.19, P>0.05, R^2^ = 0.52) and Pachycephalidae (F_1,1_ = 1.36, P>0.05, R^2^ = 0.58) displayed positive relationships that were not significant. Finally, Petroicidae was divided into the two species that make up this family and both showed a positive but statistically non-significant relationship of parasite prevalence to temperature: *Tregellasia capito* (F_1,1_ = 33.22, P>0.05, R^2^ = 0.97); and *Heteromyias albispecularis* (F_1,1_ = 14.74, P>0.05, R^2^ = 0.88).

Testing relationships of temperature to prevalence for each genus of parasite showed that prevalence of the predominant parasite *Haemoproteus* was positively related to temperature (F_1,4_ = 37.621, P<0.003, R^2^ = 0.90). Relationships for *Leucocytozoon* (F_1,4_ = 4.90, P<0.09, R^2^ = 0.55), *Trypanosoma* (F_1,4_ = 4.45, P<0.1, R^2^ = 0.53) and *Plasmodium* (F_1,4_ = 0.54, P<0.5, R^2^ = 0.12) were also positive but were not statistically significant.

### Seasonal Changes of Parasite Prevalence

The positive relationship between parasite prevalence and temperature held even when the data were divided into monthly averages (F_1,10_ = 14.44, P<0.003, R^2^ = 0.59; Fig 2B), but the regression explained less of the variation than the mean values of parasite prevalence and annual temperature. Estimates of parasite prevalence during the dry season (May-November) tended to be higher than expected under the linear model, while wet season (December-April) were lower than expected (Figure 2B). We also evaluated the relationship between monthly parasite prevalence and rainfall and found no relationship (F_1,10_ = 1.43, P<0.02, R^2^ = 0.04). The multiple regression model including both independent variables (monthly temperature and rainfall) to predict parasite prevalence was significant (F_2,9_ = 10.238, P<0.005, R^2^ = 0.69, Adjusted R^2^ = 0.63 ) but again only temperature contributed significantly to the model (temperature Beta  = 0.76 P = 0.003; rainfall Beta  = −0.32 P>0.11).

**Figure 3:**
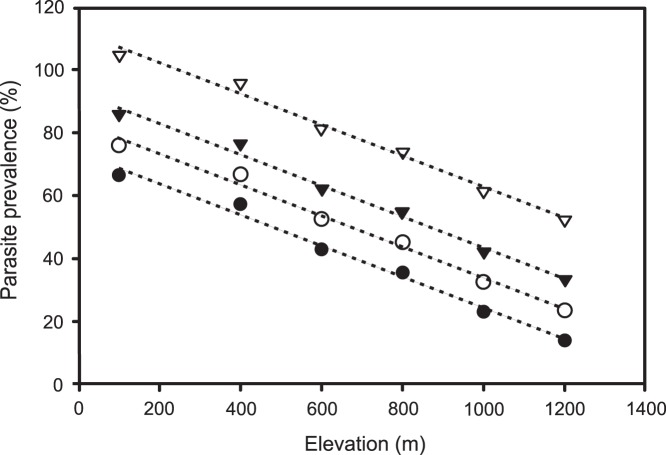
Extrapolations of parasite prevalence with increments of temperature. Parasite prevalence along the elevational gradient with increments of 0°C (•), 1°C (○), 2°C (▴) and 4°C (Δ), using the equation of the linear regression between overall parasite prevalence and mean annual temperature (temperature –140.62/0.1047 =  parasite prevalence). Extrapolations indicated that there will be an increase of about 10% in the prevalence of parasites for each 1°C of increment in temperature.

## Discussion

We found strong relationships of temperature to overall parasite prevalence. To facilitate the discussion, we used the lowland (0–400 m) and the upland (600–1200 m) distinction of climatic zones, based on forest structure [31]. In general, birds inhabiting the lowland areas where temperatures were higher had higher parasite prevalence, whereas species distributed in the upland regions with lower temperatures had lower parasite prevalence. There were similar trends for each genus of parasites surveyed. Results for lineage diversity showed that the four genera of parasites were generalist. However, most of the *Haemoproteus* lineages were partially specific to host family. This supports the approach of analysing parasite distribution within each well-sampled family separately. Prevalence within each family and within the two well sampled species showed the same trends along the gradient as for overall parasite prevalence, showing that the decrease in prevalence with elevation did not reflect a changing composition of host taxa with elevation.

**Figure 4:**
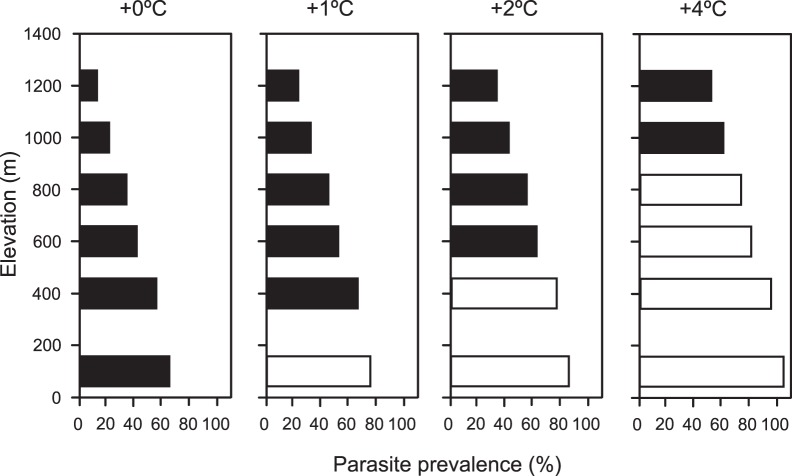
Elevational shifts upwards of bird distributions. One of the mechanisms proposed to compensate increments of parasite prevalence at 0, 1, 2 and 4°C increase in temperature. Filled bars represent the predicted distribution of birds with increments of temperature. At 0°C all bars are filled representing the actual distribution of birds along the elevation gradient, with prevalence variation from 64% in the lowlands to 16% at the highest elevations. For each 1°C increase in temperature, bird distributions need to ascend 200 m in elevation in order to avoid an increase in parasite prevalence. Open bars indicate that birds at that site shifted upwards to the next elevation site to avoid an increase in parasite prevalence, leaving that site unoccupied. Failure to make such a distribution shift would potentially result in higher mortality or reduced reproduction because of elevated blood parasite loads. The shifts in parasite loads are likely to be very large. At an altitude of 1200 m, for example, a 4°C temperature rise is predicted to increase parasite prevalence from 16% to 50%. At this higher temperature, only birds that currently live at 400 m or below will be able to offset increases in parasite prevalence by shifting their distributions upwards; for birds currently living above 400 m, some increase in parasite prevalence are unavoidable.

One of the mechanisms that could explain these results is that abundance of vectors is directly related to temperature. Bird haematozoa are transmitted by arthropod vectors [32], and ecological factors associated with vector abundance can explain differences in the prevalence of parasite species independently of host [33]–[36]. Studies in the Hawaiian islands have shown a negative correlation between abundance of mosquitoes, the main vector for *Plasmodium*, and elevation [12]. Like most vector-borne diseases, transmission of avian malaria is affected by ambient temperature. The onset, duration, and completion of the parasite’s development to the infective stage in the vector are determined by temperature. The development of *Plasmodium* occurs between 16–30°C, temperatures lower than 16°C inhibit parasite development, whereas optimal temperatures fluctuate between 28–30°C [7]. Other potential blood parasites vectors are ectoparasites that can include parasitic flies like hippoboscid flies (potential vectors for *Haemoproteus*) and sucking lice (documented vectors of filarial worms) [37], [38]. The effects of temperature and rainfall on the intensity of infection for this group of parasites are controversial. For example, a global study of current and future habitat suitability for ticks under different climate change scenarios predicts that even though some tick species are likely to undergo range expansions, others may suffer drastic range contractions worldwide [39]. Studies on parasitic flies whose larvae infect bird nestlings show the same controversial results. A study of parasitic flies of the genus *Philornis* on Argentinean forest birds found that temperature and rainfall were positively correlated with intensity of infection [40]. In contrast, another study on parasitic *Protocalliphora* (blow flies) on swallows showed that the number of blow flies varied in a curvilinear fashion with temperature, with parasite loads highest in nest around 25°C and decreasing at both higher and lower temperatures [41]. The results found in our study suggest that low temperatures of the higher elevations, especially during winter, can help to reduce both the development of avian haematozoa and the abundance of these parasite vectors, leasing to low parasite prevalence. In contrast, the high temperatures of the lowland areas provide an excellent environment for the development and transmission of haematozoa. However, further research will be vital to determine both specific vectors for each parasite genus and their trends of distribution along the elevation gradient.

The AWT are characterized by two marked seasons, the wet and dry. The dry season begins in May and is extended and acute until October or November, when the wet season begins. There was an interesting trend for parasite prevalence during the dry season to be higher, and lower during the wet season. However, we found no significant relationship between monthly parasite prevalence and rainfall. Further research is needed to show the influence of seasonal shifts that include both changes in rainfall and temperature.

### Implications for Infection Dynamics in a Warming Climate

Average global temperatures increased 0.6°C in the period 1901–2000 [42] and they are expected to increase by 1.4°C to 5.8°C by 2100 [43]. In tropical regions, this temperature increase may be accompanied by heightened variability in rainfall with more severe dry seasons [44], [45]. The regression of overall parasite prevalence and temperature documented in this study predicts an increase of about 10% in the prevalence of parasites, for each 1°C increment in temperature (Figure 3). Hosts could respond to this in three ways. First, their immune systems could adapt to the higher parasite pressure. However, the life cycles of birds are much longer than of the parasites and rapid adaptation is unlikely. Second, there could be increased mortality rates and/or lower birth rates in host populations, reducing population density. Decreased reproductive success has been associated with high infection of *Haemoproteus* and *Leucocytozoon* in passerine birds [13], [14]. *Haemoproteus* can also cause severe disease and high mortality in avian hosts [15]. Third, birds could shift their elevational distributions to hold parasite loads constant.

Figure 4 illustrates the shifts of host distribution along the elevation gradient that would be required to hold parasite prevalence to current values. Filled bars represent the predicted distribution of birds with increments of temperature. At 0°C all bars are filled representing the actual distribution of birds along the elevation gradient. For each 1°C increase in temperature, bird distributions would need to ascend 200 m in elevation. Open bars indicate that birds at that site shifted upwards to the next elevation site to avoid an increase in parasite prevalence, leaving that site unoccupied. Given a 4°C temperature increase, only birds that currently live at 400 m or below would be able to offset increases in parasite prevalence by shifting their distributions upwards; therefore for birds currently living above 400 m, some increase in parasite prevalence would be unavoidable. In Hawaii, study of the availability of disease-free habitat with increments of 2°C found that there will be a reduction of the low-disease habitat and predicted that high-elevation forest will be the most important areas to preserve the low risk disease habitat [46].

The predicted increase of parasite prevalence due to increased temperature could interact with, and further exacerbate, the projected impacts of decreased range size, increased fragmentation, and decreased population size of birds due to climate change; all these can lead to an increased risk of extinction, specially for species inhabiting the uplands [20], [21]. Our results show that upland areas are currently a low-disease habitat and their conservation must be given high priority in the management plans under climate change. Suggestions for future research include the study of intensity of infection to identify trends along the gradient.

## Supporting Information

Table S1 **The full list of frequency of detection of blood parasites.** *Haemoproteus* (*Hae*), *Plasmodium* (*Pla*), *Leucocytozoon* (*Leu*) and *Trypanosoma* (*Try*) in all the avian species presented alphabetically by family. Number of infected individuals/number of individuals sampled are shown.
(DOC)

Table S2 **Primer sequences used for the two PCR step reactions to detect blood** **parasites.** Primers used to amplify Cytochrome b (Cyt-b) in Plasmodium (Pla), Haemoproteus (Hae) and Leucocytozoon (Leu), and 18 S rRNA (18 S) in Trypanosoma (Try).
(DOC)

Table S3 **The full list of parasite lineages and host species.** MalAvi lineage names (http://mbio-serv4.mbioekol.lu.se/avianmalaria), GenBank accession numbers, Parasite genus, Host Family and Host species.
(DOC)

Table S4 **Regressions between Parasite prevalence and host ecological variables.** Relationships between Parasite prevalence and host: a) Geographic range size, b) Body mass and c) Body size. All regressions are low and none significant.
(DOC)
