# Supplementary material for: Environmental Temperature Affects Prevalence of Blood Parasites of Birds on an Elevation Gradient: Implications for Disease in a Warming Climate
Source: PLoS One. 2012 Jun 19;7(6):e39208. doi: 10.1371/journal.pone.0039208 (PMC3378574; doi:10.1371/journal.pone.0039208)
Supplement: Table S1 — The full list of frequency of detection of blood parasites. Haemoproteus (Hae), Plasmodium (Pla), Leucocytozoon (Leu) and Trypanosoma (Try) in all the avian species presented alphabetically by family. Number of infected individuals/number of individuals sampled are shown. (DOC) [file pone.0039208.s001.doc]

| **Infected species** | **No.** | **Parasite** | | | | |
| --- | --- | --- | --- | --- | --- | --- |
| **by host** | **infected/sampled** |  |  | **Unknown** |  |  |
|  |  | ***Hae*** | ***Pla*** | **(*Pla* and/or *Hae*)** | ***Leu*** | ***Try*** |
| **ACANTHIZIDAE** |  |  |  |  |  |  |
| *Sericornis keri* | 1/27 | 0 | 0 | 0 | 1 | 0 |
| *Gerygone mouki* | 1/2 | 0 | 0 | 0 | 0 | 1 |
| *Gerygone palpebrosa* | 2/2 | 2 | 0 | 0 | 0 | 0 |
| *Oreoscopus gutturalis* | 1/15 | 0 | 1 | 0 | 0 | 0 |
| *Sericornis magnirostris* | 10/25 | 7 | 1 | 1 | 0 | 1 |
| *Acanthiza katherina* | 0/11 | 0 | 0 | 0 | 0 | 0 |
| *Sericornis citreogularis* | 1/29 | 0 | 0 | 0 | 0 | 1 |
| **ALCEDINIDAE** |  |  |  |  |  |  |
| *Alcedo azurea* | 0/2 | 0 | 0 | 0 | 0 | 0 |
| **CLIMACTERIDAE** |  |  |  |  |  |  |
| *Cormobates leucophaeus* | 0/2 | 0 | 0 | 0 | 0 | 0 |
| **COLUMBIDAE** |  |  |  |  |  |  |
| *Chalcophaps indica* | 0/3 | 0 | 0 | 0 | 0 | 0 |
| **DICAEIDAE** |  |  |  |  |  |  |
| *Dicaeum hirundinaceum* | 3/3 | 0 | 0 | 0 | 3 | 3 |
| **DICRURIDAE** |  |  |  |  |  |  |
| *Rhipidura fuliginosa* | 1/7 | 0 | 0 | 0 | 0 | 1 |
| *Rhipidura rufifrons* | 1/14 | 0 | 0 | 1 | 0 | 0 |
| *Monarcha trivirgatus* | 7/18 | 1 | 0 | 0 | 7 | 0 |
| *Machaerirhynchus flaviventer* | 4/4 | 3 | 0 | 0 | 0 | 4 |
| **ESTRILDIDAE** |  |  |  |  |  |  |
| *Erythrura trichroa* | 1/17 | 0 | 0 | 1 | 0 | 0 |
| *Neochmia temporalis* | 3/31 | 1 | 0 | 1 | 2 | 0 |
| **EUPETIDAE** |  |  |  |  |  |  |
| *Psophodes olivaceus* | 0/1 | 0 | 0 | 0 | 0 | 0 |
| **MELIPHAGIDAE** |  |  |  |  |  |  |
| *Lichenostomus frenatus* | 1/10 | 1 | 0 | 0 | 0 | 0 |
| *Myzomela obscura* | 2/2 | 1 | 0 | 0 | 0 | 1 |
| *Acanthorhynchus tenuirostris* | 1/18 | 0 | 0 | 1 | 0 | 0 |
| *Meliphaga gracilis* | 1/3 | 0 | 1 | 0 | 0 | 0 |
| *Meliphaga lewinii* | 2/19 | 0 | 0 | 0 | 2 | 0 |
| *Xanthotis macleayana* | 5/7 | 0 | 0 | 3 | 5 | 1 |
| *Myzomela sanguinolenta* | 1/1 | 1 | 0 | 0 | 0 | 0 |
| *Phylidonyris nigra* | 0/6 | 0 | 0 | 0 | 0 | 0 |
| *Meliphaga notata* | 2/2 | 0 | 1 | 0 | 0 | 1 |
| **MUSCICAPIDAE** |  |  |  |  |  |  |
| *Zoothera lunulata* | 0/1 | 0 | 0 | 0 | 0 | 0 |
| **NECTARINIIDAE** |  |  |  |  |  |  |
| *Nectarinia jugularis* | 1/1 | 0 | 0 | 1 | 0 | 0 |
| **PACHYCEPHALIDAE** |  |  |  |  |  |  |
| *Colluricincla boweri* | 6/14 | 6 | 0 | 0 | 0 | 1 |
| *Pachycephala pectoralis* | 1/6 | 6 | 0 | 0 | 0 | 1 |
| *Pachycephala simplex* | 1/2 | 0 | 1 | 0 | 0 | 1 |
| *Colluricincla megarhyncha* | 6/10 | 4 | 0 | 1 | 0 | 2 |
| **PARADISAEIDAE** |  |  |  |  |  |  |
| *Ptiloris victoriae* | 3/3 | 3 | 0 | 0 | 3 | 1 |
| **PETROICIDAE** |  |  |  |  |  |  |
| *Heteromyias albispecularis* | 39/51 | 31 | 1 | 5 | 0 | 7 |
| *Tregellasia capito* | 17/22 | 17 | 0 | 0 | 0 | 1 |
| **PTILONORHYNCHIDAE** |  |  |  |  |  |  |
| *Ailuroedus melanotis* | 2/4 | 0 | 0 | 0 | 2 | 0 |
| *Prionodura newtoniana* | 0/2 | 0 | 0 | 0 | 0 | 0 |
| *Scenopoeetes dentirostris* | 0/1 | 0 | 0 | 0 | 0 | 0 |
| **ZOSTEROPIDAE** |  |  |  |  |  |  |
| *Zosterops lateralis* | 3/5 | 2 | 1 | 0 | 0 | 0 |
| **TOTAL** | 131/403 | 80 | 7 | 15 | 25 | 28 |

**Table S1. The full list of frequency of detection of blood parasites**

*Haemoproteus* (*Hae*), *Plasmodium* (*Pla*), *Leucocytozoon* (*Leu*) and *Trypanosoma* (*Try*) in all the avian species presented alphabetically by family. Number of infected individuals/number of individuals sampled are shown.
