# Supplementary material for: Environmental Temperature Affects Prevalence of Blood Parasites of Birds on an Elevation Gradient: Implications for Disease in a Warming Climate
Source: PLoS One. 2012 Jun 19;7(6):e39208. doi: 10.1371/journal.pone.0039208 (PMC3378574; doi:10.1371/journal.pone.0039208)
Supplement: Table S2 — Primer sequences used for the two PCR step reactions to detect blood parasites. Primers used to amplify Cytochrome b (Cyt-b) in Plasmodium (Pla), Haemoproteus (Hae) and Leucocytozoon (Leu), and 18 S rRNA (18 S) in Trypanosoma (Try). (DOC) [file pone.0039208.s002.doc]

| **Gene** | **Step** | **Target genera** | **Primer name** | **Primer sequence** | **Ref** |
| --- | --- | --- | --- | --- | --- |
| Cyt-b | 1^st^ | *Pla, Hae, Leu* | HaemNFI | 5’-CATATATTAAGAGAAITATGGAG-3’ | [1] |
|  |  |  | HaemNR3 | 5’-ATAGAAAGATAAGAAATACCATTC-3’ |  |
|  | 2^nd^ | *Pla, Hae* | HaemF | 5’-ATGGTGCTTTCGATATATGCATG-3’ | [2] |
|  |  |  | HaemR2 | 5’-GCATTATCTGGATGTGATAATGGT-3’ |  |
|  | 2^nd^ | *Leu* | HaemFL | 5’-ATGGTGTTTTAGATACTTACATT-3’ | [1] |
|  |  |  | HaemR2L | 5’-CATTATCTGGATGAGATAATGGIGC-3’ |  |
| 18S | 1^st^ | *Try* | S762 | 5’-GACTTTTGCTTCCTCTA(A/T)TG-3’ | [3] |
|  |  |  | S763 | 5’-CATATGCTTGTTTCAAGGAC-3’ |  |
|  | 2^nd^ | *Try* | S755 | 5’-CTACGAACCCTTTAACAGCA-3’ | [3] |
|  |  |  | S823 | 5’-CGAA(T/C)AACTGC(C/T)CTATCAGC-3’ |  |

**Table S2*.* Primer sequences used for the two PCR step reactions to detect blood**

**parasites**

Primers used to amplify Cytochrome b (Cyt-b) in *Plasmodium* (*Pla*), *Haemoproteus* (*Hae*) and *Leucocytozoon* (*Leu*), and 18S rRNA (18S) in *Trypanosoma* (*Try*). Universal base inosine (I)

1. Hellgren O, Waldenstrom J, Bensch S (2004) A new PCR assay for simultaneous studies of Leucocytozoon, Plasmodium, and Haemoproteus from avian blood. Journal of Parasitology 90: 797-802.

2. Bensch S, Stjernman M, Hasselquist D, Ostman O, Hansson B, et al. (2000) Host specificity in avian blood parasites: a study of Plasmodium and Haemoproteus mitochondrial DNA amplified from birds. Proceedings of the Royal Society of London Series B-Biological Sciences 267: 1583-1589.

3. Sehgal RNM, Jones HI, Smith TB (2001) Host specificity and incidence of Trypanosoma in some African rainforest birds: a molecular approach. Molecular Ecology 10: 2319-2327.
