# Supplementary material for: Environmental Temperature Affects Prevalence of Blood Parasites of Birds on an Elevation Gradient: Implications for Disease in a Warming Climate
Source: PLoS One. 2012 Jun 19;7(6):e39208. doi: 10.1371/journal.pone.0039208 (PMC3378574; doi:10.1371/journal.pone.0039208)
Supplement: Table S3 — The full list of parasite lineages and host species. MalAvi lineage names (http://mbio-serv4.mbioekol.lu.se/avianmalaria), GenBank accession numbers, Parasite genus, Host Family and Host species. (DOC) [file pone.0039208.s003.doc]

|  | **MalAvi** | **GenBank** | **Parasite** | **Host Family** | **Host species** |
| --- | --- | --- | --- | --- | --- |
|  | **lineage** | **accession** | **genus** |  |  |
|  | **name** | **number** |  |  |  |
| 1 | TRECAP02 | JX021535 | Haemoproteus | Petroicidae | *Heteromyias albispecularis* |
| 2 | GERPAL01 | JX021536 | Haemoproteus | Acanthizidae | *Gerygone palpebrosa* |
| 3 | NEOTEM01 | JX021537 | Haemoproteus | Estrildidae | *Neochmia temporalis* |
| 3 | NEOTEM01 | JX021537 | Haemoproteus | Dicruridae | *Monarcha trivirgatus* |
| 4 | PTIVIC01 | JX021538 | Haemoproteus | Paradisaeidae | *Ptiloris victoriae* |
| 5 | TRECAP03 | JX021539 | Haemoproteus | Petroicidae | *Tregellasia capito* |
| 6 | TRECAP04 | JX021540 | Haemoproteus | Petroicidae | *Tregellasia capito* |
| 7 | PTIMAG01 | JX021541 | Haemoproteus | Paradisaeidae | *Ptiloris victoriae* |
| 8 | PTIVIC02 | JX021542 | Haemoproteus | Paradisaeidae | *Ptiloris victoriae* |
| 8 | PTIVIC02 | JX021542 | Haemoproteus | Dicruridae | *Machaerirhynchus flaviventer* |
| 9 | TRECAP06 | JX021543 | Haemoproteus | Petroicidae | *Tregellasia capito* |
| 10 | HETALB04 | JX021544 | Haemoproteus | Petroicidae | *Heteromyias albispecularis* |
| 11 | TRECAP07 | JX021545 | Haemoproteus | Petroicidae | *Tregellasia capito* |
| 12 | HETALB02 | JX021546 | Haemoproteus | Petroicidae | *Heteromyias albispecularis* |
| 13 | TRECAP01 | JX021547 | Haemoproteus | Petroicidae | *Tregellasia capito* |
| 13 | TRECAP01 | JX021547 | Haemoproteus | Petroicidae | *Heteromyias albispecularis* |
| 14 | COLMEG02 | JX021548 | Haemoproteus | Pachycephalidae | *Colluricincla megarhyncha* |
| 14 | COLMEG02 | JX021548 | Haemoproteus | Pachycephalidae | *Colluricincla boweri* |
| 15 | HETALB01 | JX021549 | Haemoproteus | Petroicidae | *Heteromyias albispecularis* |
| 16 | ZOSLAT04 | JX021550 | Haemoproteus | Zosteropidae | *Zosterops lateralis* |
| 17 | TRECAP08 | JX021551 | Haemoproteus | Petroicidae | *Tregellasia capito* |
| 18 | GERPAL02 | JX021552 | Haemoproteus | Acanthizidae | *Gerygone palpebrosa* |
| 19 | SERCIT02 | JX021553 | Haemoproteus | Acanthizidae | *Sericornis magnirostris* |
| 20 | LICFRE03 | JX021554 | Haemoproteus | Meliphagidae | *Lichenostomus frenatus* |
| 21 | MYZSAN01 | JX021555 | Haemoproteus | Meliphagidae | *Myzomela sanguinolenta* |
| 21 | MYZSAN01 | JX021555 | Haemoproteus | Meliphagidae | *Myzomela obscura* |
| 22 | HETALB03 | JX021556 | Haemoproteus | Petroicidae | *Heteromyias albispecularis* |
| 23 | SERCIT01 | JX021557 | Haemoproteus | Acanthizidae | *Sericornis magnirostris* |
| 23 | SERCIT01 | JX021557 | Haemoproteus | Acanthizidae | *Gerygone mouki* |
| 24 | HETALB05 | JX021558 | Haemoproteus | Petroicidae | *Heteromyias albispecularis* |
| 25 | HETALB06 | JX021559 | Haemoproteus | Petroicidae | *Heteromyias albispecularis* |
| 26 | HETALB07 | JX021560 | Haemoproteus | Petroicidae | *Heteromyias albispecularis* |
| 27 | HETALB08 | JX021561 | Haemoproteus | Petroicidae | *Heteromyias albispecularis* |
| 28 | TRECAP09 | JX021562 | Haemoproteus | Petroicidae | *Tregellasia capito* |
| 29 | TRECAP10 | JX021563 | Haemoproteus | Petroicidae | *Tregellasia capito* |
| 30 | ZOSLAT05 | JX021564 | Haemoproteus | Zosteropidae | *Zosterops lateralis* |
| 31 | MELNOT02 | JX021565 | Plasmodium | Meliphagidae | *Meliphaga notata* |
| 32 | ZOSLAT06 | JX021566 | Plasmodium | Zosteropidae | *Zosterops lateralis* |
| 33 | FANTAIL01 | JX021567 | Plasmodium | Petroicidae | *Heteromyias albispecularis* |
| 33 | FANTAIL01 | JX021567 | Plasmodium | Pachycephalidae | *Pachycephala simplex* |
| 33 | FANTAIL01 | JX021567 | Plasmodium | Acanthizidae | *Sericornis magnirostris* |
| 34 | OREGUT01 | JX021568 | Plasmodium | Acanthizidae | *Oreoscopus gutturalis* |
| 35 | MELGRA01 | JX021569 | Plasmodium | Meliphagidae | *Meliphaga gracilis* |
| 36 | XANMAC01 | JX021570 | Leucocytozoon | Meliphagidae | *Xanthotis macleayana* |
| 37 | XANMAC02 | JX021571 | Leucocytozoon | Meliphagidae | *Xanthotis macleayana* |
| 38 | MELLEW02 | JX021572 | Leucocytozoon | Meliphagidae | *Meliphaga lewinii* |
| 39 | AILMEL01 | JX021573 | Leucocytozoon | Ptilonorhynchidae | *Ailuroedus melanotis* |
| 40 | PTIVIC03 | JX021574 | Leucocytozoon | Paradisaeidae | *Ptiloris victoriae* |
| 41 | PTIVIC04 | JX021575 | Leucocytozoon | Paradisaeidae | *Ptiloris victoriae* |
| 42 | HEAL01 | JX021576 | Trypanosoma | Petroicidae | *Heteromyias albispecularis* |
| 43 | HEAL02 | JX021577 | Trypanosoma | Petroicidae | *Heteromyias albispecularis* |
| 43 | HEAL02 | JX021577 | Trypanosoma | Paradisaeidae | *Ptiloris victoriae* |
| 43 | HEAL02 | JX021577 | Trypanosoma | Dicruridae | *Machaerirhynchus flaviventer* |
| 44 | RHFU01 | JX021578 | Trypanosoma | Dicruridae | *Rhipidura fuliginosa* |
| 45 | SEMA01 | JX021579 | Trypanosoma | Acanthizidae | *Sericornis magnirostris* |
| 45 | SEMA01 | JX021579 | Trypanosoma | Petroicidae | *Heteromyias albispecularis* |
| 45 | SEMA01 | JX021579 | Trypanosoma | Petroicidae | *Tregellasia capito* |
| 45 | SEMA01 | JX021579 | Trypanosoma | Pachycephalidae | *Colluricincla megarhyncha* |
| 45 | SEMA01 | JX021579 | Trypanosoma | Pachycephalidae | *Colluricincla boweri* |
| 45 | SEMA01 | JX021579 | Trypanosoma | Meliphagidae | *Xanthotis macleayana* |
| 46 | MENO01 | JX021580 | Trypanosoma | Meliphagidae | *Meliphaga notata* |
| 47 | PAPE01 | JX021581 | Trypanosoma | Pachycephalidae | *Pachycephala pectoralis* |
| 47 | PAPE01 | JX021581 | Trypanosoma | Pachycephalidae | *Pachycephala simplex* |
| 48 | HEAL03 | JX021582 | Trypanosoma | Petroicidae | *Heteromyias albispecularis* |

## Table S3. The full list of parasite lineages and host species

MalAvi lineage names (<http://mbio-serv4.mbioekol.lu.se/avianmalaria> ), GenBank accession numbers, Parasite genus, Host Family and Host species are indicated.
