# Supplementary material for: Environmental Temperature Affects Prevalence of Blood Parasites of Birds on an Elevation Gradient: Implications for Disease in a Warming Climate
Source: PLoS One. 2012 Jun 19;7(6):e39208. doi: 10.1371/journal.pone.0039208 (PMC3378574; doi:10.1371/journal.pone.0039208)
Supplement: Table S4 — Regressions between Parasite prevalence and host ecological variables. Relationships between Parasite prevalence and host: a) Geographic range size, b) Body mass and c) Body size. All regressions are low and none significant. (DOC) [file pone.0039208.s004.doc]

| **Variable** | **F** | ***P*** | **R^2^** |
| --- | --- | --- | --- |
| 1. Range size | (1,16)=1.59 | 0.23 | 0.09 |
| 1. Body mass | (1,16)=4.02 | 0.06 | 0.20 |
| 1. Body size | (1,16)=2.45 | 0.14 | 0.13 |
|  | | | |

**Table S4.** **Regressions between Parasite prevalence and host ecological**

**variables**

Relashionships between Parasite prevalence and host: a) Geographic range size, b)Body mass and c) Body size. All regressions are low and none significant.
